# Bifunctional Organic Polymeric Catalysts with a Tunable Acid-Base Distance and Framework Flexibility

**DOI:** 10.1038/srep06475

**Published:** 2014-09-30

**Authors:** Huanhui Chen, Yanan Wang, Qunlong Wang, Junhui Li, Shiqi Yang, Zhirong Zhu

**Affiliations:** 1 Department of Chemistry, Tongji University, Shanghai 200092, China

## Abstract

Acid-base bifunctional organic polymeric catalysts were synthesized with tunable structures. we demonstrated two synthesis approaches for structural fine-tune. In the first case, the framework flexibility was tuned by changing the ratio of rigid blocks to flexible blocks within the polymer framework. In the second case, we precisely adjusted the acid-base distance by distributing basic monomers to be adjacent to acidic monomers, and by changing the chain length of acidic monomers. In a standard test reaction for the aldol condensation of 4-nitrobenzaldehyde with acetone, the catalysts showed good reusability upon recycling and maintained relatively high conversion percentage.

Heterogeneous acid-base bifunctional catalysts, activating both electrophilic and nucleophilic reactants, have gained a great deal of attention because of their biomimetic nature^1^,^2^,^3^,^4^,^5^,^6^. These catalysts maintain antagonistic acidic and basic groups in a compatible coexistence that is not achievable in homogeneous systems. Compared with artificial catalysts, enzymes have relatively complex spatial architectures. The distance between functional groups within the enzymatic structure prevents incompatible functional groups from mutually quenching each other and allows activated substrates to approach one another. Furthermore, slight modification of the distances between acidic and basic sites can have a significant impact on catalytic performance^7^,^8^,^9^,^10^,^11^,^12^,^13^,^14^,^15^. The induced-fit hypothesis assumes that in general the enzymatic framework is partially flexible. During binding, the substrate causes a conformational change in the enzyme it associates with, such that the active sites achieve the exact configuration required for a particular reaction to occur^16^. To effectively mimic this aspect of enzyme function, the preparation of bifunctional catalysts with precisely controlled framework flexibility and finely tuned distances between different functionalities is necessary.

Previous work on bifunctional solid catalysts has focused on the co-localization of acidic and basic sites on mesoporous silica supports^17^,^18^,^19^,^20^,^21^,^22^,^23^. Brunelli and Didas reported different cooperative interactions between amino functionalities and silanols by varying the alkyl chain length of organosilane molecules randomly grafted onto SBA-15 mesoporous silica supports^24^. Zeidan et al. investigated the cooperative effect between amino groups and acids of varying strengths^23^. However, neither precise adjustment of the distance between a given acid and amino group nor successful tuning of a flexible catalyst framework supported on mesoporous silica has been reported in the literature. In contrast to mesoporous silica-based bifunctional catalysts that lack well-designed morphologies, organic Polymeric materials are promising candidates for biomimetic catalysis because of their framework flexibility, structural compositions that are similar to those of the enzyme, structural controllability, wide availability and the diversity of raw materials that can be used in their synthesis.

In this work, we developed a simple and general method for preparing acid-base bifunctional polymeric catalysts. The flexibility of the framework can be tuned by changing the ratio of rigid blocks to flexible blocks that comprise the polymer (Figure 1a). The distance between the acidic and basic sites can be adjusted precisely either by distributing the basic monomer to be adjacent to the acidic monomer units or by altering the linker length of the acidic monomers (Figure 1b). By keeping the amount of basic monomer **3** and acidic monomer **4a** constant and varying the amount of styrene **5a** from 0 to 7 equivalents, we synthesized catalysts with a variety of flexible backbones and used them to evaluate the relationship between framework flexibility and catalytic activity. It should be noted that poly [(*N*-4-vinylbenzyl butylamine)-co-(acrylic acid)] (**Poly[2-co-4]**) is insoluble in acetone, whereas polystyrene is soluble; hence, the blocks of **Poly[2-co-4]** and polystyrene are rigid and flexible, respectively. Using an array of catalysts synthesized with acrylic acid and acrylyl-amino acids as acidic monomers with different chain length, we investigated the effects of the distance between the acidic and basic sites on the catalytic performance of the aldol condensation of 4-nitrobenzaldehyde and acetone.

## Results and Discussion

### Formation of catalysts

**Figure 1:**
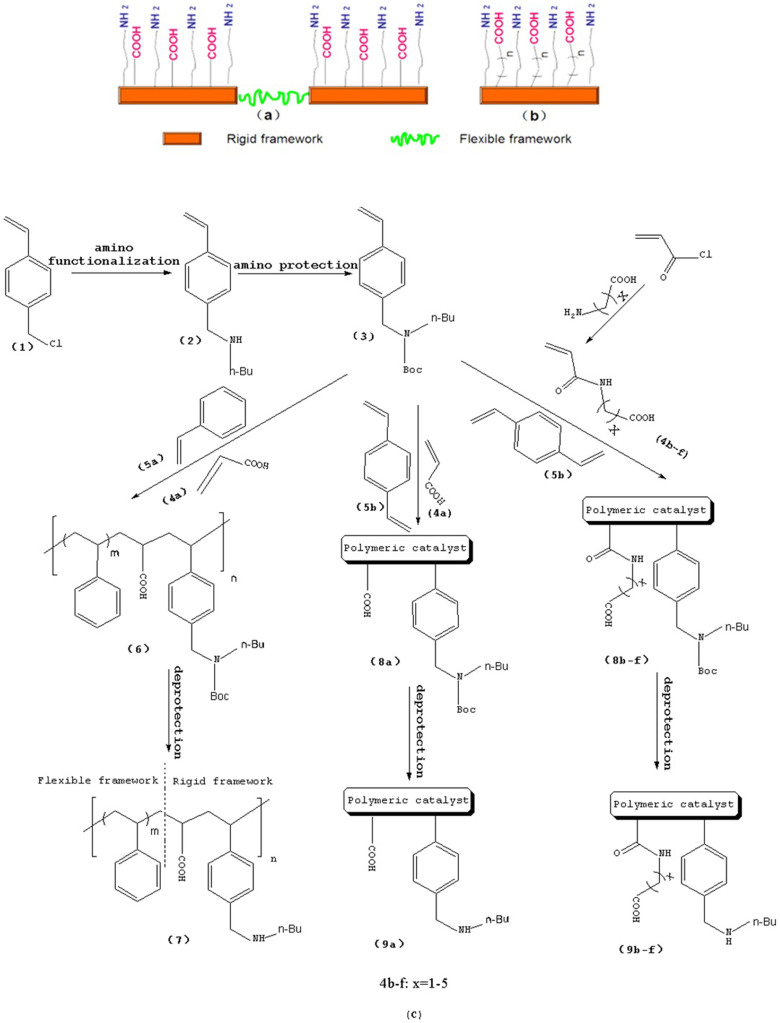
(a) Schematic of the framework flexibility tuning process. (b) Schematic of tuning the distance between acid and base sites. (c) Synthesis of bifunctional organic catalysts.

A detailed description of the steps in the bifunctional catalyst synthesis, material characterization, and catalytic activity testing can be found in the Supporting Information, but a brief account is provided below (Figure 1c).

First, **1** was reacted with n-butylamine to give **2**, which was used as the basic monomer in the synthesis^25^. Then, the amino group of this monomer unit was protected using di-tert-butyl-dicarbonate, a method commonly used for amino group protection, giving **3**^26^,^27^. Acryloyl chloride was reacted with amino acids in aqueous solution to obtain the corresponding acidic monomer (**4a**–**f**)^28^. **6** was obtained by combining monomers **3**,**4a** and **5a** through a radical polymerization reaction. **8(a**–**f)** were obtained using monomers **3**,**4(a**–**f)** and **5b**. Deprotection of the amino group by thermal treatment yielded **7 (**or **9a**–**f)**. In this way, the catalytically active sites of the catalyst were separated, and an organic polymeric sample containing two antagonistic functional groups was obtained.

The intermediate **6 (**or **8a**–**f)** and the final bifunctional catalyst **7 (**or **9a**–**f)** were characterized by ^13^C NMR, ^13^C solid-state NMR, FTIR spectroscopy, ninhydrin coloring and titration. The spectra of **6** and **8** as well as those of **7** and **9** were similar to one another, and as such, we compared only those of **6** and **7**.

### Characterization of catalysts

**Figure 2:**
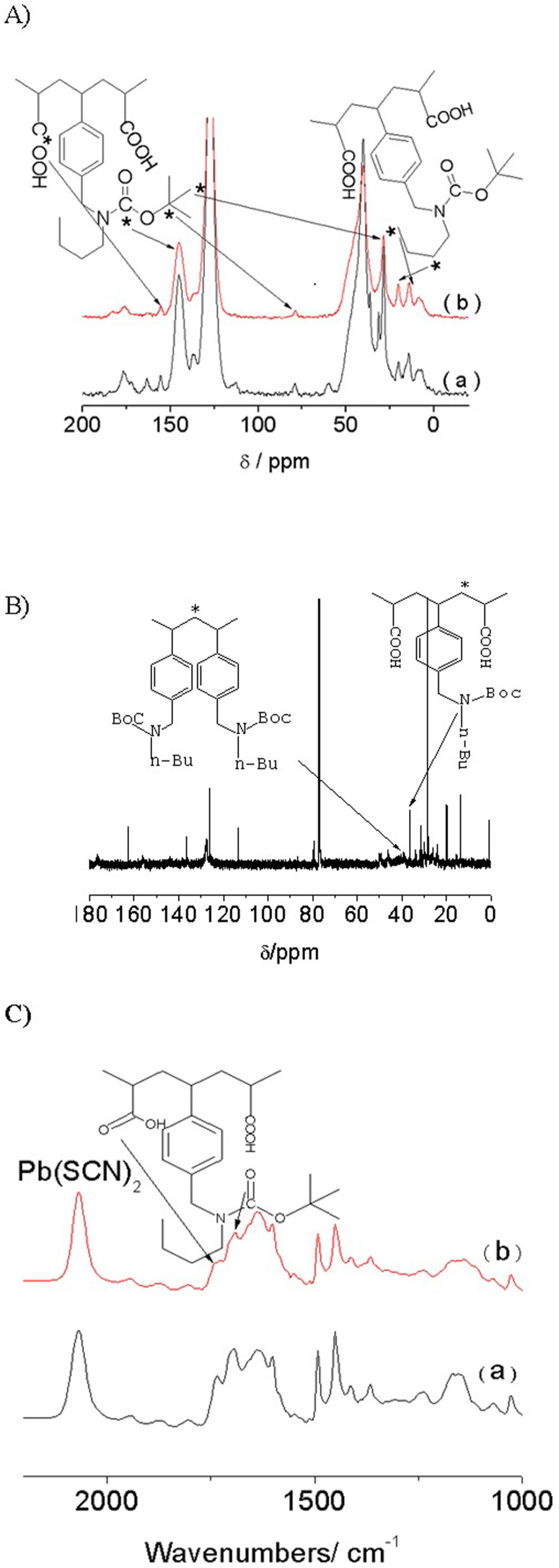
(A) ^13^C CP-MAS NMR spectra of 6 and 7, and (B) ^13^C NMR spectra of 6, where * marks the C resulting in the corresponding peak.(C) FTIR spectra of 6 and 7, where Pb(SCN)_2_ is used as the internal standard.

The successful cohabitation of acidic and basic sites in **6** and **7** was confirmed by NMR spectroscopy (Figure 2A). The ^13^C solid-state NMR spectrum of **6** shows resonances at δ = 176.6 (COOH), 28.0 ((CH_3_)_3_-C), 78.6 (C-O), 13.0 (CH_3_), 20.0 (CH_2_), 46.2 (Ar-C-N and C-COOH), 134.0 (Ar) and 157.1 ppm (C = O). These shifts demonstrate that monomers **3**, **4a** and **5a** participated in the polymerization reaction. In the ^13^C solid-state NMR spectrum of **7**, the weakening of the Boc signals at δ = 28.0, 78.6 and 157.1 ppm indicated that part of carbamate had decomposed into the amino functionality. Furthermore, the resonances associated with the carboxyl group decreased because some of carboxyl groups were removed along with the deprotection of amino group.

To obtain an orderly distribution of acid and base sites, the large degree of steric hindrance inherent in basic monomer **3** was exploited to reduce its homopolymerization. In the ^13^C NMR spectrum of **6** (Figure 2B), δ = 36.5 ppm was assigned to the CH_2_ of the adjacent acid-base blocks. There is no signal at δ = 40 ppm attributed to the CH_2_ in the homopolymerization of the basic monomers. This indicates that the basic monomer resides exclusively adjacent to the acidic monomer after synthesis.

The successful incorporation of carboxyl groups and amino groups in as-synthesized catalysts was further confirmed by FTIR spectroscopy (Figure 2C). The FTIR spectra of **6** and **7** display characteristic peaks at 1745 cm^−1^ assigned to the CO vibrations of carboxyl groups, 1699 cm^−1^, which is attributed to the CO vibration of carbamate, and 2925–3059 cm^−1^, ascribed to aromatic CH vibrations.

The extent of deprotection of the amino groups in **7** was also determined by ninhydrin color tests (Figure 1 in SI). **7** turned violet in the ninhydrin solution, whereas the color of **6** did not change. This result leads to the conclusion that free amino groups exist in the structure of **7**.

The amount of acid and base present in the catalyst structure was quantitatively determined by titration. It was found that **7** contains approximately 0.27 mmol g^−1^ carboxylic acid and 0.25 mmol g^−1^ amine, respectively; the titration results of the other synthesis intermediates and products are listed in Table 1 in the SI.

### Effect of framework flexibility on catalysis

The catalyst **7** was utilized in the aldol condensation of 4-nitrobenzaldehyde with acetone (Table 1), a standard test reaction, to assess the degree of cooperativity in the prepared acid-base bifunctional catalysts^29^,^30^,^31^,^32^.

The framework flexibility had a significant impact on the catalytic performance, as shown in Table 1. In the table, it is seen that the observed conversion rate first increased and then decreased with increasing framework flexibility. In the two extreme cases, very rigid framework (catalyst 7-0) and very flexible framework (catalyst 7-6 and 7-7) were both unfavorable for the catalyst performance. We found that only when the ratio of flexible blocks to rigid blocks was in the range of 2:1 to 3:1, then the rate of conversion reached a maximum of ~93%. It is interesting to see that catalyst with the most flexible framework (7-7) did not show any catalysis performance. In this case, the framework experienced too much flexibility (flexible blocks:rigid blocks = 8:1) which resulted in functional groups self neutralization. We believe that in catalysts 7-1 and 7-2, the right degree of flexibility within the framework may have facilitated two processes so as to enhance conversion: (1) the flexible framework allowed the group of activated substrates to approach each other; and (2) the flexible catalysts changed conformation and forced the substrates into strained or distorted structures that resembles the transition state of the reaction. Another interesting phenomenon as can be seen in Table 1 is the selectivity of the catalysts. In catalysts 7-1 and 7-2, which all gave ~93% of total conversion, the ratio of product A to product B was very different, being about 1:6 and 1:2, respectively. This suggests that polymer-based catalysts with tunable framework flexibility may in the near future be used to control reaction pathway and tune the product ratio.

### Effect of the acid-base distance on catalytic activity

It has been confirmed with ^13^C NMR spectrum that the basic monomer resides exclusively adjacent to the acidic monomer on the framework. In that case, if both of amino and carboxyl side chain are flexible, they would lean to each other by electrostatic attraction, as such, amine was protonated^33^. To adjust acid-based distance precisely, we used arylamine as a rigid side chain and acrylyl-amino acids as a flexible side chain with controllable chain length. Because rigid side chain cannot lean towards carboxy group,only when the flexible linker is long enough, the neutralization may then occur (Figure 4 in SI).

**Figure 3:**
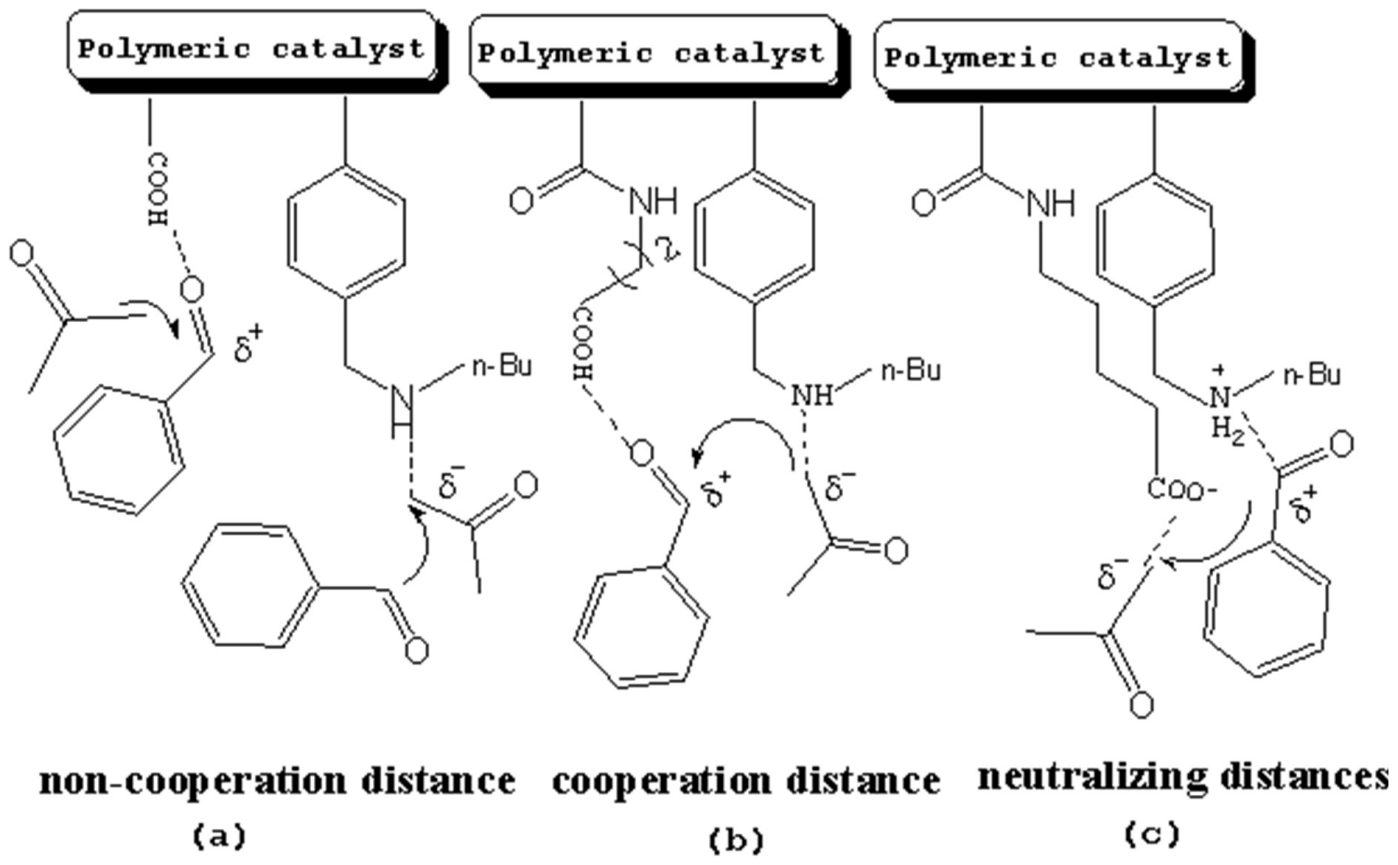
The relationship between acid-base distance and catalytic performance.

It is expected that different distances between amino and carboxyl groups would confer different catalytic activities for the bifunctional catalysts. To investigate this possibility, the catalytic performances of bifunctional catalysts **9a**–**f** were tested. In each of these catalysts, the distance between acidic and basic sites was tuned by utilizing carboxylic acids with different chain lengths. The catalytic results are shown in Table 2. The catalyst made using the shortest chain acid (**9a**) was found to catalyze aldol condensation with the lowest conversion, 13.3%, indicating that the acrylic acid chain is too short for effective cooperative interactions to occur (Figure 3a). The activated substrates in this case could only react with free reactants, which were consequently inactivated. When the acid monomer of the catalyst was acrylyl-glycine (**9b**), the conversion sharply increased by more than six-fold to approximately 79.5%. Acrylyl-β-alanine (**9c**) produced a further increase in the conversion rate (~81.5%). It can be concluded, then, that upon the incorporation of this acidic monomer, the optimal acid-base distance for aldol condensation was achieved (Figure 3b). These last two catalysts prevented the acidic and basic sites from undergoing mutual quenching and allowed the activated substrates to approach one another and react. Furthermore, the activation energy of the reaction when performed over these materials was reduced significantly. The catalysts that incorporated long chain acids, namely **9d**, **9e** and **9f,** led to the conversions of 51.9%, 48.2% and 43.3%, respectively. This result suggested that the acid sites on **9d**, **9e**, and **9f,** synthesized with acrylyl-γ-aminobutyric acid (**4d**), acrylyl-5-Aminovaleric acid (**4e**) or acrylyl-6-aminocaproic acid (**4f**), were close to the basic sites and that, for this reason, neutralization occurred. The weaker bases (carboxylic ion) and weaker acids (ammonium ion) exhibited a lower catalytic efficiency (Figure 3c). We deduce, thus, that the optimal distance between the acidic and basic sites of a bifunctional catalyst is 1 to 2 bond lengths when used for the aldol condensation of 4-nitrobenzaldehyde with acetone.

### Catalyst-quenching experiments

To probe the bifunctionality of polymeric catalysts, 8a was treated with HCl to neutralize the amine groups, thus leaving only the carboxylic acid (Table 3, entry2). Likewise, 8a (Table 3, entry3) was treated with ammonia which gave a resulting material whose most acid sites were neutralized. These results illustrate the bifunctionality and the coexistent acidity and basicity. Use of **8a** with either carboxyl acid or amine functional group preserved on it gave markedly low conversion levels (Table 3, entries 2 and 3). Furthermore, when **8a** with only acid groups was physically mixed with **8a** with only base groups, the mixture possessed an intermediate conversion level that was much lower than those of the bifunctional acid-base catalysts tested (Table 3, entry 1). These experiments highlight the properties achievable by immobilization of bifunctional groups. When the heterogeneous catalyst 8a is neutralized with homogeneous acids or bases, the opposing functional group salted out and the respective catalytic behavior was lost. On the contrary, the two opposing groups did not lose catalytic activity when carboxylic acid and the amine are immobilized on polymer but rather functioned cooperatively to obtain the most active catalyst.

### Comparison with mesoporous silica material

Compared with mesoporous silica catalysts which is higher acid density and surface area but not well designed-structure^18^,^24^,^32^,^33^,^34^,^35^, the bifunctional organic polymeric catalysts exhibited more or less equal conversion rate and higher 4-(4-Nitrophenyl)-3-buten-2-one (**B**) selectivity. The higher selectivity of **B** is because the stronger carboxylic acid is more effective in catalyzing the dehydration of **A** than the weaker bronsted acid silanol. Besides, the controlable structure could be also the reason of high selectivity.

### Reusability of the Catalyst

The reusability of the catalyst 9b was tested using aldol condensation as the model reaction. After the completion of one cycle, the catalyst was filtered from the reaction system, thoroughly washed with acetone, dried under vacuum at 130°C for 2h to adequately remove absorbed water, then used in the next cycle. The fresh bifunctional catalyst gave 80% conversion. In the second run, the conversion slightly dropped to 73% under the same reaction condition. After the second cycle the conversion becomes steady in the following cycles (for example, 73% conversion for the ninth cycle), which means the catalysts are stable and could be recycled.

### The application in other reactions

In addition to the above mentioned model reaction, our catalyst could be also applied in Michael Addition, Henry Reaction, and Knoevenagel Condensation reaction, which all exhibited good performance. The experimental results are given in the SI section.

## Conclusions

In summary, acid-base bifunctional organic polymeric catalysts with tunable structure were successfully synthesized for the first time. The innovative catalyst structural tuning methods reported in this paper originated from the idea of biomimicity of protein catalytic reaction. In contrast to previously reported catalysts, the current materials can be precisely tuned in terms of both the distance between the acidic and basic sites and the flexibility of the polymeric catalyst framework. By carefully changing the ratio of rigid blocks and flexible blocks within the polymer framework, we found that the framework flexibility has a significant impact on catalytic performance. Maximum catalytic conversion was observed only within a certain range of flexibility. In several test experiments, we also found that the optimal distance between the acidic and basic sites should be between 1 and 2 bond lengehs to efficiently catalyze the aldol condensation of 4-nitrobenzaldehyde and acetone. As an extension of this work, we are currently investigating the potential of making imprint of substrate molecules within the bifunctional catalysts in order to fit the spatial structure of the catalyst to the substrate more precisely.

## Methods

### Catalytic experiments

Catalyst was added (0.05 mmol total amine) to a solution of 4-nitrobenzaldehyde (76 mg, 0.5 mmol) in acetone (10 mL), and the reaction flask was then sealed under nitrogen and heated at 50°C for 20 h. Acetone was then removed in vacuo, and the product was analyzed by ^1^HNMR spectroscopy in CDCl_3_ with THF as the internal standard.

## Supplementary Material

Supplementary Information Supplementary Information

## Figures and Tables

**Table 1:** Effect of framework flexibility on catalysis

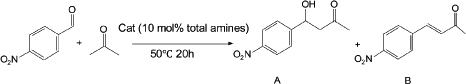					
Entry	Catalyst 7-n^[a]^	R^[b]^	A[%]	B[%]	Conv. [%]^[c]^
**1**	**7-0**	0	19	7	26
**2**	**7-1**	1	14	79	93
**3**	**7-2**	2	28	65	93
**4**	**7-3**	3	28	48	76
**5**	**7-4**	4	26	43	69
**6**	**7-5**	5	23	29	52
**7**	**7-6**	6	9	16	25
**8**	**7-7**	7	0	0	0

[a] n is the weight-based ratio of styrene and basic monomer.

[b]R is the ratio of flexible blocks to rigid blocks/

[c] Total conversion. Yields determined through ^1^H NMR spectroscopic analysis.

**Table 2:** Effect of acid-base distance on catalysis

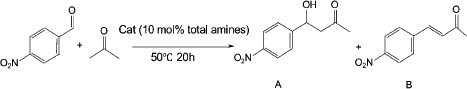				
Entry	Catalyst	A[%]	B[%]	Conv[%]^a^
**1**	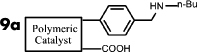	5	9	14
**2**	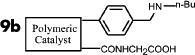	23	57	80
**3**	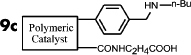	23	58	82
**4**	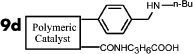	23	30	52
**5**	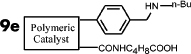	21	27	48
**6**	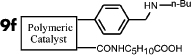	18	26	43

[a] Total conversion. Yields determined by ^1^H NMR spectroscopic analysis.

**Table 3:** Catalyst-quenching experiments

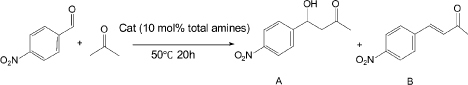				
Entry	Catalyst	A[%]	B[%]	Conv[%]^a^
**1**	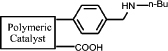	5	9	14
**2**	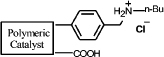	trace	trace	trace
**3**	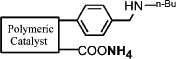	2	1	3
**4**	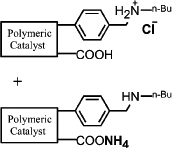	3	4	7

[a] Total conversion. Yields determined by ^1^H NMR spectroscopic analysis.
