# Supplementary material for: Bifunctional Organic Polymeric Catalysts with a Tunable Acid-Base Distance and Framework Flexibility
Source: Sci Rep. 2014 Sep 30;4:6475. doi: 10.1038/srep06475 (PMC5377424; doi:10.1038/srep06475)
Supplement: Supplementary Information [file srep06475-s1.doc]

Bifunctional Organic Polymeric Catalysts with Tunable Acid-base Gap and Framework Flexibility

*Huanhui Chen, Yanan Wang, Qunlong Wang, Junhui Li, Shiqi Yang,and Zhirong Zhu**

Department of chemistry, Tongji University, Shanghai 200092 ,China

**Corresponding Author**

*Email: zhuzhirong@tongji.edu.cn

**Characterization**

FT-IR spectra were collected with a Nicolet Nexus 470 IR spectrometer with KBr pellet. 13C (100.5 MHz) cross-polarization magic angle spinning (CP-MAS) solid-state NMR experiments were recorded on a Varian infinity-plus 400 spectrometer equipped with a magic-angle spin probe in a 4 mm ZrO_2_ rotor. Experimental parameters for ^13^C CP-MAS NMR experiments: 8-kHz spin rate, 3-s pulse delay, 4-min contact time, 1500-3000 scans. ^1^H NMR spectra were obtained at 400 MHz and recorded relative to the tetramethylsilane signal (0 ppm) or residual protio-solvent. ^13^C NMR spectra were obtained at 100 MHz, and chemical shifts were recorded relative to the solvent resonance (CDCl_3_, 77.0 ppm).

**Experimental details：**

All glassware was oven or flame dried immediately prior to use. All solvents were purified and dried according to standard methods prior to use. All reagents were obtained from commercial sources and used without further purification, unless stated otherwise.

**Synthesis of N-(4-Vinylbenzyl)butylamine (2)：**

4-Vinylbenzylchloride (5.00 g, 32.8 mmol) and n-butylamine (23.99 g, 328 mmol) were placed in a 250 mL round bottomed flask and the mixture stirred for 24 hours at room temperature. The excess of n-butylamine was removed in a rotary evaporator. The resulting slurry was dispersed in diethyl ether to precipitate the salt formed in the reaction which was removed by filtration. The remaining salts were removed by extraction with water/diethyl ether (×3). The organic phase was dried over MgSO_4_ and the solvent removed. The product was purified by distillation to give a yellow oil (yield 90% yield).[1]

**Synthesis of Butyl-(4-vinyl-benzyl)-carbamic acid tert-butyl ester (3):**

Boc_2_O (2.29 g, 10.5 mmol) in CH_2_Cl_2_ (10 ml) was added to a soluton of compound 1 (1.89 g, 1.0 mmol) in CH_2_Cl_2_ (10 ml) at 10°C, and  the resulting  mixture  was  stirred at room temperature for 2 h. The solvent was removed in vacuo, and the residue was purified by silica gel column chromatography to afford the corresponding pure 3. ^1^H NMR (400 MHz, CDCl_3_) δ 7.36 (d, J = 8.1 Hz, 2H), 7.19 (d, J = 7.8 Hz, 2H), 6.75 – 6.65 (q, 1H), 5.77 – 5.69 (d, 1H), 5.22 (d, J = 10.9 Hz, 1H), 4.45 – 4.36 (s, 2H), 3.15 (s, J = 0.4 Hz, 2H), 1.46 (s, 11H), 1.32 – 1.22 (m, 2H), 0.89 (t, J=7.3, 3H).^13^CNMR (100 MHz, CDCl3) δ 155.89, 136.56, 133.93, 129.49, 127.71, 126.32, 113.56, 79.51, 49.86, 46.24, 30.17, 28.48, 20.05, 13.86.

**Synthesis of acrylyl-**[amino](app:ds:amino) [acid](app:ds:acid)**s (4b-f):**

acrylyl-[amino](app:ds:amino) [acid](app:ds:acid)s monomers were synthesized from the following materials, C_1_: Glycine; C_2_: 3-aminopropanoic acid; C_3_: 4-aminobutyric acid; C_4_: 5-aminovaleric acid; C_5_: 6-aminocaproic acid. Brieﬂy, 0.1 mol glycine and 0.11 mol NaOH were dissolved in 80 ml deionized water in an ice bath under vigorous stirring. To this, 0.11 mol acryloyl chloride in 15 ml tetrahydrofuran was added drop wise. The pH was maintained at 7.5-7.8 until the reaction was complete. The reaction mixture was then extracted using ethyl acetate. The clear aqueous layer was acidiﬁed to pH 2.0 and then extracted with ethyl acetate. The organic layers were collected, combined, and dried over sodium sulfate. The solution was then ﬁltered, concentrated, and precipitated in petroleumether. Further puriﬁcation was achieved by repeated precipitation, and the ﬁnal product was lyophilized. Synthesis of other monomers followed similar procedure, with variations in pH during the acidiﬁcation: pH 2.0 for C_2_, and C_3_; pH 3.0 for C_4_, C_5_, and C_6_ [2].

**Synthesis of Poly[styrene-co-(Butyl-(4-vinyl-benzyl)-carbamic acid tert-butyl ester)-co-( acrylic acid)] (6):**

0 g, 0.28 g, 0.56 g,0.84 g ,1.12 g 1.40 g,1.68g or 1.96 g styrene (purified according to standard methods prior to use). was added to a solution of AIBN（0.01g）, 3 (0.28g) and 4a (0.072g) respectively. the solution was then stirred at 85℃under the atmosphere of nitrogen for 4h,giving an array of framework [flexibility](app:ds:flexibility) 6.

**Synthesis of Poly[divinyl benzene-co-(Butyl-(4-vinyl-benzyl)-carbamic acid tert-butyl ester)-co-( acrylic acid)] (8a) :**

0.01g AIBN was added to a solution of 0.92 mmol divinylbenzene (purified according to standard methods prior to use), 1 mmol 3 and 1 mmol**4a** in 3 ml DMF. the solution was stirred at 85℃ under the atmosphere of nitrogen for 6 h. The resulting jelly was then extracted with petroleum ether (400 mL per gram) by refluxing in petroleum ether for 24 h to remove the residual monomers. The solid was then allowed to dry in vacuo.

**Synthesis of Poly [divinyl benzene-co-(Butyl-(4-vinyl-benzyl)-carbamic acid tert-butyl ester)-co-(acrylyl-**[amino](app:ds:amino) [acid](app:ds:acid)**)] (8b-f) :**

0.01g AIBN was added to a solution of 0.92 mmol divinylbenzene (purified according to standard methods prior to use), 1mmol 3 and 1mmol**4b-f** in 3ml DMF. the solution was stirred at 85℃ under the atmosphere of nitrogen for 6 h.This solution was added slowly to vigorously stirred copious amounts of water at 0℃. The precipitate was filtered and dried in vacuo, to afford 8b-f respectively

**Synthesis of 7 ( 8a-f ) :**

0.5 g of solid sample was introduced into a 100 mL one-necked round bottom flask. The flask was heated at 150 ℃ under vacuum for 20 h.

**Catalytic experiments:**

**Aldol condensation.** Catalyst was added (0.05 mmol totalamines) to a solution of 4-nitrobenzaldehyde (76 mg, 0.5 mmol) in acetone (10 mL), and the reaction flask was then sealed under [nitrogen](app:ds:nitrogen) and heated at 50℃ for 20 h. Acetone was then removed in vacuo, and the product was analyzed by ^1^HNMR spectroscopy in CDCl_3_ with THF as an internal standard.

**Michael Addition.** A typical reaction was conducted using about 10 mg of catalyst (the amount of catalyst was fixed at 0.02 molar equivalents of amine relative to trans-β-nitrostyrene) in 8 mL of an anhydrous benzene solution of concentration 0.022 M in trans-β-nitrostyren and 0.044 M of malononitrile. The reaction was performed at room temperature, analyzed by gas chromatography using 1,3,5-trimethoxybenzene as an internal standard. Conversion and selectivity were determined via^1^H NMR by taking aliquots via syringe filter and diluting with CDCl_3_. Resonances: 5.6ppm (β-nitro alcohol product), 7.5 ppm (nitrobenzene), 7.72-7.74 ppm (α, β-unsaturated product), and 10.1 ppm (4-nitrobenzaldehyde).

**Henry Reaction.** Reactions were conducted using approximately 30 mg of catalyst (the amount of catalyst was fixed at 0.01 molar equivalents of amine relative to 4-nitrobenzaldehyde).To this vessel was added a solution containing 5 mmol nitromethane, 0.5 mmol 4-nitrobenzaldehyde, and 0.05 mmol nitrobenzene as an internal standard. The reaction was performed at 40°C. Conversion and selectivity were determined via^1^H NMR by taking aliquots via syringe filter and diluting with CDCl_3_. Resonances: 5.6 ppm (β-nitro alcohol product), 7.5 ppm (nitrobenzene), 7.72-7.74 ppm (α, β-unsaturated product), and 10.1 ppm (4-nitrobenzaldehyde).

**Knoevenagel Condensation.** A typical reaction was conducted using about 10 mg of catalyst (the amount of catalyst was fixed at 0.01 molar equivalents of amine relative to 3-nitrobenzaldyde) in 8 mL of an anhydrous benzene solution of concentration 0.022 M in 3-nitrobenzaldyde and 0.044 M of malononitrile. The reaction was performed at room temperature, and aliquots were taken by syringe and analyzed by gas chromatography using 1,3,5-trimethoxybenzene as an internal standard.

**Ninhydrin colouring:**

6a or 7a (0.02g) were added to 0.2% ethanol solution of ninhydrin (2ml), and then heated at 80℃ for 15 min.

**Titration:**

0.1g sample was added to 20ml HCl solution (ca 0.01M), then stirred for 0.5h, filtered, washed with large amount of distilled water. To combined solution of the filtrates and washings was added 5-6 drops of 1% Phenolphthalein solution as indicator and titrated with 0.01M NaOH solution until a faint pink colour is obtained and last for 0.5 min, calculates consumption of hydrochloricacid to get the content of basic sites.

0.1g sample was added to 20mL NaOH solution (ca 0.01M) , then stirred for 0.5h, filtered, washed with large amount of distilled water. To combined solution of the filtrates and washings was added 5-6 drops of 1% Phenolphthalein solution as indicator and titrated with 0.01 M HCl solution until a faint pink colour faded and last for 0.5 min, calculates consumption of hydrochloricacid to get the content of acidic sites.

[1]L. M. [García-Con](http://www.ncbi.nlm.nih.gov/pubmed?term=García-Con LM%5BAuthor%5D&cauthor=true&cauthor_uid=20506437) , M. J. Whitcombe , E.V Piletska, S. A. [Piletsky,](http://www.ncbi.nlm.nih.gov/pubmed?term=Piletsky SA%5BAuthor%5D&cauthor=true&cauthor_uid=20506437) *Angew. Chem. Int. Ed*. **2010**, *49*, 4075.

[2]R. [Ayala](http://www.ncbi.nlm.nih.gov/pubmed?term=Ayala R%5BAuthor%5D&cauthor=true&cauthor_uid=21396708), C. [Zhang](http://www.ncbi.nlm.nih.gov/pubmed?term=Zhang C%5BAuthor%5D&cauthor=true&cauthor_uid=21396708), D. [Yang](http://www.ncbi.nlm.nih.gov/pubmed?term=Yang D%5BAuthor%5D&cauthor=true&cauthor_uid=21396708) , Y. Hwang, A. Aung, *Biomaterials*. **2011**, *32*, 3700


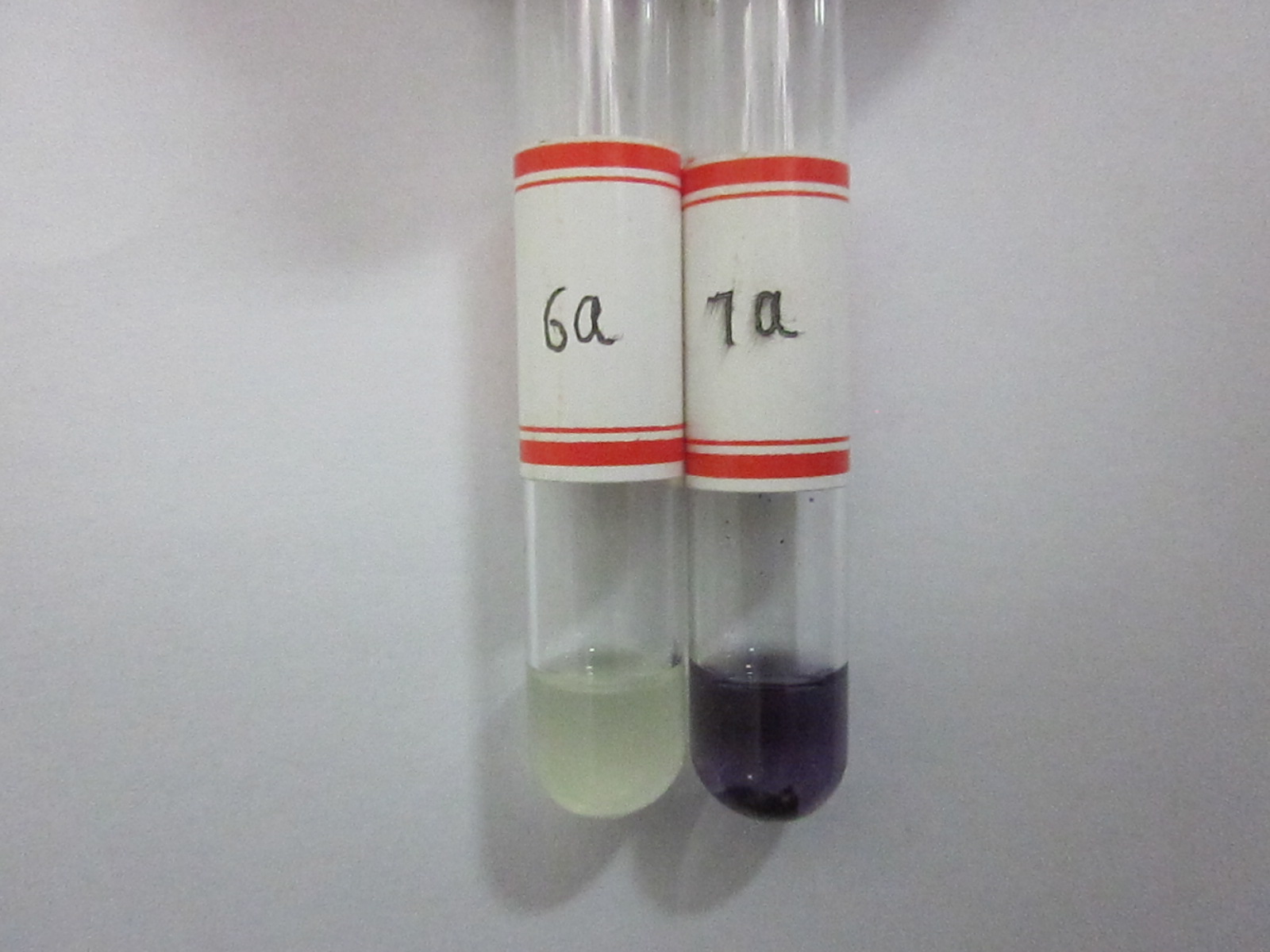


***Figure 1.*** Ninhydrin colouring of 7a


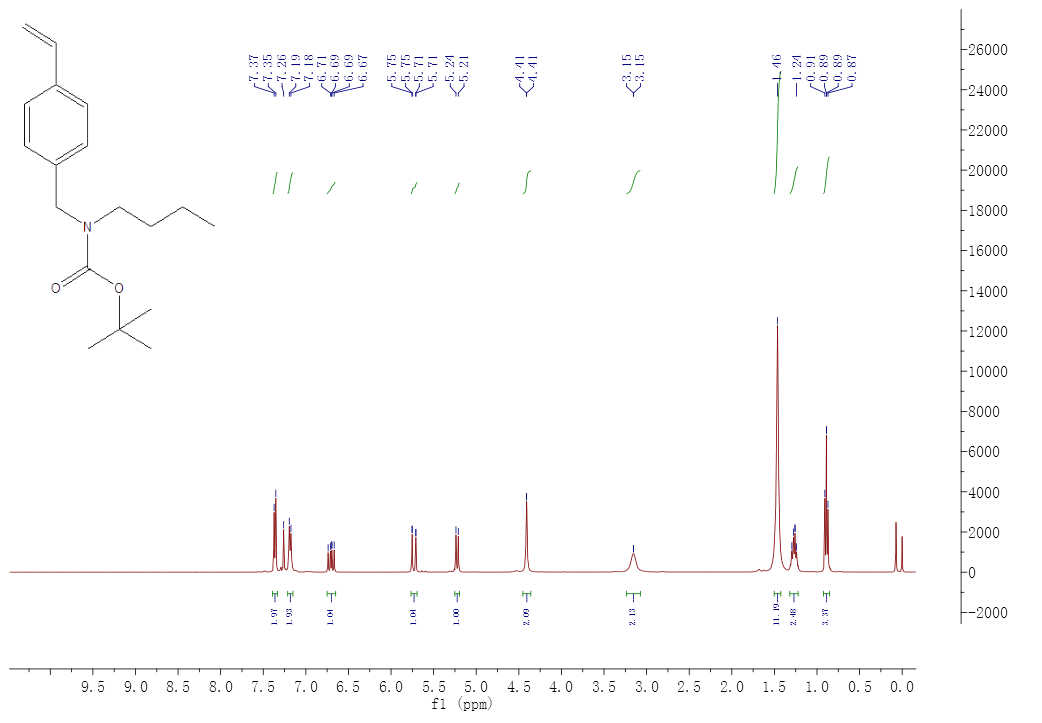


***Figure 2.*** ^1^H NMR Spectra of 3


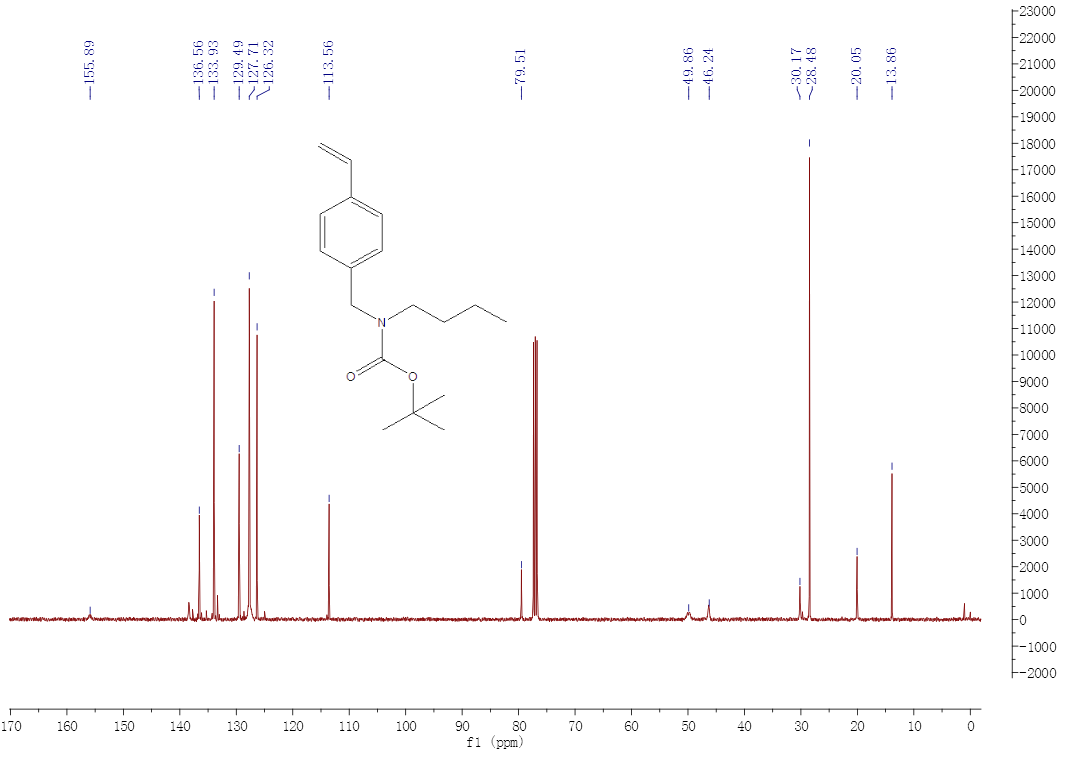


***Figure 3.*** ^13^C NMR Spectra of 3

***Figure 4. the effect of the flexiblity of side chain on the distance***

***Table 1．****The acid and base content of each catalyst*

| content | 7-0 | 7-1 | 7-2 | 7-3 | 7-4 | 7-5 | 7-6 | 7-7 | 8a | 8b | 8c | 8d | 8e | 8f |
| --- | --- | --- | --- | --- | --- | --- | --- | --- | --- | --- | --- | --- | --- | --- |
| Acid  content(mmol/g) | 0.36 | 0.27 | 0.20 | 0.18 | 0.15 | 0.13 | 0.11 | 0.08 | 0.27 | 0.25 | 0.23 | 0.23 | 0.22 | 0.20 |
| Base content(mmol/g) | 0.37 | 0.27 | 0.19 | 0.17 | 0.14 | 0.11 | 0.10 | 0.07 | 0.25 | 0.24 | 0.22 | 0.21 | 0.21 | 0.19 |

**Table 2．The applications in Michael Addition, Henry Reaction, Knoevenagel Condensation**

| **Entry** | **Catalyst** | **Michael Con** | **Knoevenagel Con** | **Henry Con** |
| --- | --- | --- | --- | --- |
| **1**  **2**  **3** |  | 39.8%  trace  27.4% | ＞99%  trace  52.7% | 89.3%  trace  54.3% |
